# Global COVID-19 Policy Engagement With Scientific Research Information: Altmetric Data Study

**DOI:** 10.2196/46328

**Published:** 2023-06-29

**Authors:** Han Woo Park, Ho Young Yoon

**Affiliations:** 1 Department of Media & Communication YeungNam University Gyeongsan-si Republic of Korea; 2 Graduate Department of Digital Convergence Business and East Asian Cultural Studies YeungNam University Gyeongsan-si Republic of Korea; 3 Cyber Emotions Research Center YeungNam University Gyeongsan-si Republic of Korea; 4 Big Local Big Pulse Lab YeungNam University Gyeongsan-si Republic of Korea; 5 Division of Communication & Media Ewha Womans University Seoul Republic of Korea

**Keywords:** altmetrics, government policy report, citation analysis, COVID-19, World Health Organization, WHO, COVID-19 research, online citation network, policy domains

## Abstract

**Background:**

Previous studies on COVID-19 scholarly articles have primarily focused on bibliometric characteristics, neglecting the identification of institutional actors that cite recent scientific contributions related to COVID-19 in the policy domain, and their locations.

**Objective:**

The purpose of this study was to assess the online citation network and knowledge structure of COVID-19 research across policy domains over 2 years from January 2020 to January 2022, with a particular emphasis on geographical frequency. Two research questions were addressed. The first question was related to who has been the most active in policy engagement with science and research information sharing during the COVID-19 pandemic, particularly in terms of countries and organization types. The second question was related to whether there are significant differences in the types of coronavirus research shared among countries and continents.

**Methods:**

The Altmetric database was used to collect policy report citations of scientific articles for 3 topic terms (COVID-19, COVID-19 vaccine, and COVID-19 variants). Altmetric provides the URLs of policy agencies that have cited COVID-19 research. The scientific articles used for Altmetric citations are extracted from journals indexed by PubMed. The numbers of COVID-19, COVID-19 vaccine, and COVID-19 variant research outputs between January 1, 2020, and January 31, 2022, were 216,787, 16,748, and 2777, respectively. The study examined the frequency of citations based on policy institutional domains, such as intergovernmental organizations, national and domestic governmental organizations, and nongovernmental organizations (think tanks and academic institutions).

**Results:**

The World Health Organization (WHO) stood out as the most notable institution citing COVID-19–related research outputs. The WHO actively sought and disseminated information regarding the COVID-19 pandemic. The COVID-19 vaccine citation network exhibited the most extensive connections in terms of degree centrality, 2-local eigenvector centrality, and eigenvector centrality among the 3 key terms. The Netherlands, the United States, the United Kingdom, and Australia were the countries that sought and shared the most information on COVID-19 vaccines, likely due to their high numbers of COVID-19 cases. Developing nations, although gaining quicker access to COVID-19 vaccine information, appeared to be relatively isolated from the enriched COVID-19 pandemic content in the global network.

**Conclusions:**

The global scientific network ecology during the COVID-19 pandemic revealed distinct types of links primarily centered around the WHO. Western countries demonstrated effective networking practices in constructing these networks. The prominent position of the key term “COVID-19 vaccine” demonstrates that nation-states align with global authority regardless of their national contexts. In summary, the citation networking practices of policy agencies have the potential to uncover the global knowledge distribution structure as a proxy for the networking strategy employed during a pandemic.

## Introduction

### Research Background

Given the expanding international scientific linkages, it is advantageous to view this expansion as a communications network and recognize the network as a new form of global governance. This view has been made possible by the increasing worldwide scientific connections. Over the past 2 decades, the global network has grown denser and more concentrated, indicating the presence of numerous subconnections that can form exclusive cliques [1]. The expansion has occurred despite the overall reduction in the size of the network, suggesting the presence of power dynamics in global health research and development governance.

Previously, ecological studies of COVID-19 research articles were typically reviewed without considering the contextual and spatial features of the scientific system. Studies typically concentrated on the bibliometric characteristics of COVID-19 research articles [2,3], neglecting the identification of institutional actors who cite recent scientific contributions related to COVID-19 in the policy domain, and their locations. In other words, the global collaboration involved in COVID-19 research has not been exhaustively investigated in terms of the policy-related proportion of overall scientific information exchange, which is assessed by the number of citations to peer-reviewed published works.

Although scientific research publications were not the study focus, a recent study examined the institutional responses of about 300 different intergovernmental organizations to the COVID-19 epidemic [4]. This study discovered that 52.8% of intergovernmental organizations shared third-party knowledge or information, whereas only 32.5% developed their own expertise. The study was able to quantify intergovernmental organization responses to pandemics by searching the websites of global and regional organizations for references to COVID-related phrases. The study aimed to establish the government’s skills in information intervention for risk management, identify influential agents in a global information network, and discover socioeconomic elements that influence the government’s information-sharing behaviors across regions. The researchers of the study examined the online presence of various government health agencies using network analysis. They found that European agencies had the biggest web impact in response to COVID-19, and that income inequality and gross domestic product per capita were associated with the high online visibility of government health agencies. This indicates that socioeconomic factors may play a role in predicting the government’s distribution of COVID-19 information during the pandemic. The data source used in this study was not particularly exhaustive of citations found in policy documents and websites maintained by intergovernmental organizations and national government agencies.

Thus, taking sociological institutionalist and space-related scientometrics into account [5], the goal of this study was to conduct an in-depth investigation of the institutional actors who cite the most recent scientific contributions related to topics covered by COVID-19, using novel big data analytics, namely the altmetric search and data curation technique [6]. Altmetrics examine, among other things, the influence of scientific research findings on media, documents, and other types of writings. Traditional citation tracking and evaluation rely on peer-reviewed publications since citations are regarded as a sign of research impact, whereas altmetrics involve nonacademic public behavior, such as social media. As social media make research citations more efficient than traditional venues [7], altmetrics use not only social media but also scholarly databases, such as Scopus, to evaluate research output across fields. Owing to their statistical relationship with actual research impact, altmetrics are considered as a credible alternative to standard research evaluation [8], similar to Web of Science, PubMed, and Google Scholar [9]. Quantitative analyses of research articles produced by numerous authors from various countries revealed that global research collaboration networks are continuously expanding [10] and traditional measures alone would not be sufficient.

Indeed, policy papers released by internationally famous organizations as well as nationally recognized public authorities can benefit and supplement a knowledge-sharing infrastructure reference system [4]. Consequently, it would be interesting to investigate whether developing and emerging nations have expanded their engagement in global COVID-19 science as a result of the visibility provided by an innovative approach to big data analytics. Not only COVID-19 research and development spending but also policy responses in impoverished countries could not match those in affluent countries. It is apparent that the backdrop of developing countries contributes to the difficulties of responding quickly to the pandemic. Developing countries lack sufficient fiscal capacity to assist their populations for a longer period of time when social distancing is effective to prevent the spread of COVID-19 [11]. The large number of younger populations in developing countries require economic activity to support their families, making it difficult to maintain social distancing [12]. The tradition of living together as a large family, partly due to poverty, creates intergenerational living conditions that contribute to the relatively high level of COVID-19 virus exposure among older people, while the insufficient capacity of the health care system makes treating COVID-19 patients difficult [12,13]. Indeed, developing countries lack testing kits as well as infrastructure, such as health care facilities, including equipment and personnel [13,14]. All of this contributes to the greater difficulty of applying known policy responses to COVID-19 in developing countries compared with developed countries.

In this regard, an examination of the interconnected networks involved in the geopolitical features of the scientific footprint, particularly the national distribution of citation effect, would benefit the health science community while also contributing to a better understanding of the global network of policy responses, particularly for developing countries. As a result, we investigated the knowledge-sharing reference system and addressed the possibilities of establishing a new global information order through network representation of space-related research data, as well as some future research directions.

### Related Work and Research Questions

The primary purpose of big data analytics, such as altmetrics, is to monitor rapidly expanding scientific issues in the wake of social, political, and cultural events and infectious disease outbreaks [15]. When research results become a new global focal point, the function of big data metrics is significant because it enables the measurement of social media mentions, newspaper coverage, and policy document citations [16]. Numerous large-scale data analyses have been conducted amidst the COVID-19 epidemic, for example, a study analyzed the scientific information transmission networks and news-sharing behaviors regarding COVID-19 on Twitter [17]. However, relatively little systematic research has been conducted on how global and national policymakers approach COVID-19 research topics [18].

In infodemiology investigations, policy documents are regarded as an authoritative, credible, and esteemed source that serves as a valuable information resource during pandemics. Specifically, the aggregation of online citation data can serve as a starting point for evaluating strategic approaches for health administration and public campaigns, and for this reason, network analysis and big data have been integrated into the field of research [19]. As these methods can be used to improve public health, it would be beneficial to illustrate a network pattern. This would also contribute to the creation of an efficient communication system. That said, our 2 research questions are as follows:

1. Who has been the most active in terms of policy engagement with science and research information sharing during the COVID-19 pandemic? Which policy sources, in terms of website domains and countries, provide the greatest number of research references from scholarly articles on COVID-19 subjects?
2. Are there substantial differences in the types of coronavirus research shared in each country, according to citation networks? More specifically, how is the networked structure of research information sharing organized between nations and major research topics?

## Methods

### Keywords

**Figure 1:**
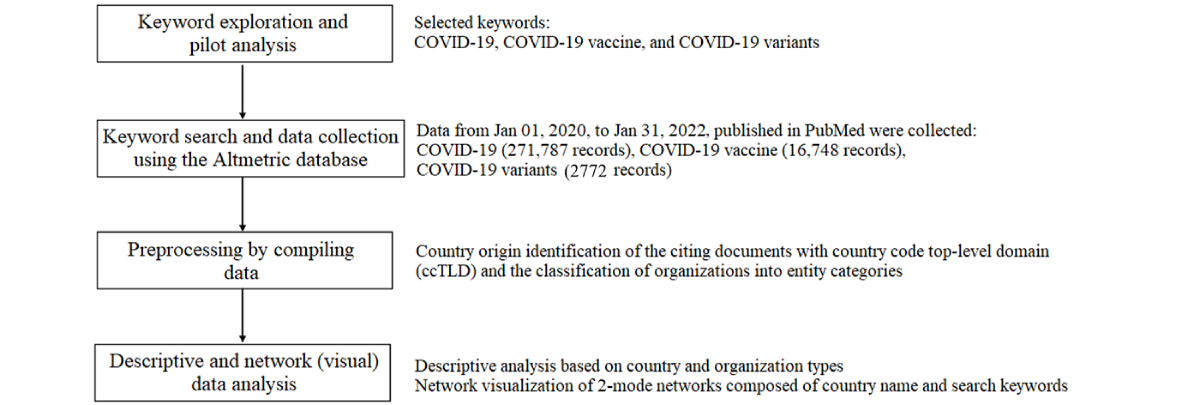
Study flowchart.

Figure 1 depicts the data collection and analysis process. The first procedure of data collection was to identify keywords that accurately represent policy contexts of the scientific research citation network. After a series of pilot studies using a range of keywords, including COVID-19 booster injection, 3 queries were ultimately selected. COVID-19, COVID-19 vaccine, and COVID-19 variants were the 3 keywords that were used.

### Data Collection and Preprocessing

The data collection procedure was as follows. First, the 3 keywords were used to extract data from the Altmetric citation database. There were 216,787 research outputs for COVID-19, 16,748 for COVID-19 vaccine, and 2772 for COVID-19 variants. These results were acquired by limiting the search to research articles published in journals indexed by PubMed between January 1, 2020, and January 31, 2022. Note that the numbers reflect the deletion of duplicate database records. As some articles appeared in both the “COVID-19” search and the “COVID-19 vaccine” or “COVID-19 variants” search, duplicate articles were removed by manually comparing the titles and author names with the Excel (Microsoft Corp) function for filters and removing duplicate records. Altmetric.com is a database of citations for both research articles and policy reports. The database includes policy sources, such as government directives, reports, and white papers, as well as publications from independent policy institutes, expert advisory committees, research institutions, and international development organizations [15]. Altmetric intends to collect global policy sources on climate change, health, transportation, and economics [19]. Typically, policy sources are updated on the websites of organizations. Altmetric derives research citations from policy documents by tracking the interest in policy sources and collecting data from organization websites. The URLs of policy agencies that cited COVID-19 studies are included in the calculation of citation attention from policy documents and texts. In general, citation frequency is used to evaluate the significance of research. Policy mentions are essential for calculating Altmetric impact scores, as citations reflect current public interest. The evaluation of policy documents relies on sources. By employing link searching, identifier analysis, text extraction, and policy mentions, Altmetric combines PDFs, metadata, and research outcomes. Through text extraction, Altmetric establishes a connection between policy document mentions and journal research. Altmetric operationalizes the national origin of policy papers that cite COVID-19 study articles by using a variety of information categories, including country code top-level domain (ccTLD), language, location, etc. Previous studies have also employed a similar strategy [20]. Altmetric provides additional information regarding the algorithm for more details.

Second, after the data set was extracted, URL information was used to determine the country of origin for policy documents. A domain is the namespace in which an organization operates, which serves as a proxy indicator of its institutional attributes and identity. Who.int, for example, refers to the World Health Organization (WHO), which is an example of an intergovernmental organization. Website visitors can determine the physical location of a domain by inspecting its ccTLD. As an illustration, the domain name researchbriefings.files.parliament.uk is clearly associated with the United Kingdom. Similarly, each URL referring to COVID-19 research publications was accompanied by website domains and the countries where they were hosted.

Third, a network data set for visualization was made. A network data set is composed of nodes and links that connect the nodes. For the citation network, nodes are research or policy documents and the links are citations between them. In other words, the governance of the COVID-19 pandemic consists of nodes (research information and policy documents) and links (citations between intergovernmental health agencies and scientific articles). By constructing the network data set, the examination of the connectedness between hosting countries and research topics resulted in the discovery of insights in the information flow.

### Analysis Methods

The process of analyzing citation networks involves quantifying the intricate and intertwined connections between the various components. This study presents an analytic approach for evaluating the competence of information intervention against COVID-19 by intergovernmental health organizations. The act of citing and disseminating research findings may be interpreted as an endeavor by public authorities to mitigate risk. The effectiveness of risk reduction strategies is evaluated based on the content of policy reports from a variety of international organizations and nations, which are referenced as URLs.

This research analyzed the data with descriptive statistics and network analysis in order to comprehend the strategies. The citing institutions were divided into 3 categories for descriptive purposes: intergovernmental organizations, national and domestic governmental organizations, and nongovernmental organizations (think tanks and academic institutions). A few basic indexes and visualization were used for network analysis. Network analysis employs statistical methods and visual mapping to investigate the configuration of nodes and their interactive connections along cited and citing dimensions [21,22]. Network analysis indicators can evaluate the structural positions and roles of health agencies and their host countries, as well as the statistical links between scientific and policy texts. Several centrality measures were used to determine the frequency of direct immediate connections in the network data sets: degree, 2-local, and eigenvector values. High centralities usually indicate how quickly nodes spread information. Central nodes transfer data to other nodes, and degree centrality counts a node’s direct links. The amount and pattern of ties are used to calculate a node’s geodesic distance and indirect fraction of direct ties. Even if a node has moderate centralities, its 2-local and eigenvector centralities may be higher because of the shortest path. Nodal centralities were measured and compared using a network diagram. UCINET-NetDraw was used to calculate and draw centralities and visualizations [23]. The network analysis results can show efficient information flow, mutual communication, or interorganizational collaboration in health policy-making institutions. Cross-national mobility of research findings can provide suggestions for public health development and promote critical junctures in cross-functional networks, as cited research articles on policy documents have become scientific intellectual settings for knowledge dissemination. Hierarchical or geographic clustering based on the interacting relationships between countries in the 3 data sets can ensure regional integration during the COVID-19 pandemic.

## Results

### Policy Engagement With Scholarly Articles

According to Table 1, the WHO was the most influential reporter in terms of citations to scientific papers. Intergovernmental organizations represented 73.5% (119/162) of citations. National and domestic governmental organizations represented the second most influential reporter. Despite the fact that COVID-19 swiftly became a pandemic, the identified institutions were not diverse. Citations are intended to serve as a guide for decision-makers, instructing them to refer to the characteristics of COVID-19 and to investigate the impact of vaccines on the effectiveness of treatment. That said, it is important to note that governmental organizations in Europe drove citations, whereas think tanks and academic institutions in the United States drove citations. Table 2 displays the distribution of countries with regard to COVID-19 citations.

**Table 1:** Website domain distribution for COVID-19 citations.

Category and website		Value (N=162), n (%)
**Intergovernmental organizations**		119 (73.5)
	who.int	108 (66.7)
	worldbank.org	4 (2.5)
	wmo.int	3 (1.9)
	europa.eu	2 (1.2)
	oecd-ilibrary.org	1 (0.6)
	fao.org	1 (0.6)
**National and domestic governmental organizations**		32 (19.8)
	folkhalsomyndighete.se	11 (6.8)
	health.gov.au	7 (4.3)
	parliament.uk	7 (4.3)
	rivm.nl	4 (2.5)
	gov.scot	2 (1.2)
	sencanada.ca	1 (0.6)
**Nongovernmental organizations (think tanks and academic institutions)**		11 (6.8)
	rand.org	5 (3.1)
	nber.org	2 (1.2)
	csis.org	2 (1.2)
	interacademies.org	1 (0.6)
	awmf.org	1 (0.6)

**Table 2:** Country distribution for COVID-19 citations.

Continent and country^a^		Value (N=162), n (%)
**Europe**		
	Switzerland	111 (68.5)
	Sweden	11 (6.8)
	United Kingdom	9 (5.6)
	Netherlands	4 (2.5)
	Belgium	2 (1.2)
	France	1 (0.6)
	Germany	1 (0.6)
	Italy	1 (0.6)
**North America**		
	United States	14 (8.6)
	Canada	1 (0.6)
**Asia/Pacific**		
	Australia	7 (4.3)

^a^The World Health Organization and World Meteorological Organization are in Switzerland, the World Bank is in the United States, the Food and Agriculture Organization is in Italy, 7 institutions of the European Union are in Belgium, and the Organization for Economic Co-operation and Development is in France.

The headquarters of the WHO is in Switzerland, and Switzerland represented 68.5% (111/162) of citations. Moreover, the United States represented 8.6% (14/162) of citations, Sweden represented 6.4% (11/162) of citations, Australia represented 4.3% (7/162) of citations, and the United Kingdom represented 5.6% (9/162) of citations. The proportion of countries other than European countries was 13.5% (22/162). Since the majority of intergovernmental bodies are located in Europe, we recalculated the values without them, and the number of citations for Europe was 25 and that for all other countries was 18. European citations were 1.40 times greater than those of other nations.

Table 3 lists the policy groups in the results on COVID-19 vaccines. The overall number was 1900, which is 11.7 times greater than the COVID-19 result of 162, suggesting that there are vested interests in vaccine effects and policy adaptation.

Regarding COVID-19 vaccines, intergovernmental organizations represented 76.5% (1453/1900) of citations, national and domestic governmental organizations represented 17.5% (333/1900) of citations, and nongovernmental organizations (think tanks and academic institutions) represented 6.0% (114/1900) of citations, similar to the findings for COVID-19 citations. For COVID-19 vaccines, the WHO represented 67.5% (1282/1900) of citations, similar to the finding for COVID-19 citations. The second most influential reporter was the Dutch government’s National Institute for Public Health and Environment (73/1900, 3.8%).

Table 4 shows the country distribution for COVID-19 vaccine citations. For COVID-19 vaccine citations, when intergovernmental organizations were included, Switzerland ranked first, followed by the Netherlands, the United States, and the United Kingdom. On the other hand, when intergovernmental organizations were excluded, the Netherlands ranked first, followed by the United States, the United Kingdom, and Australia. Ethiopia represented a noticeable number of citations (n=18), reflecting the interest of this African country in COVID-19 vaccination.

Interestingly, the Netherlands, the United States, the United Kingdom, and Australia experienced unprecedented increases in COVID-19 cases between December 2020 and January 2021. In Multimedia Appendix 1, we have presented the number of new COVID-19 cases for these 4 countries from March 2020 to March 2022. In all these countries, the number of new cases peaked around January 2021. Thus, they experienced an urgency of policy measures related to COVID-19 vaccination. Indeed, the scientific citations reflect the reality.

The number of COVID-19 variant citations was 521 (Table 5). Regarding COVID-19 variants, the WHO represented the highest number of citations (446/521, 85.6%), followed by folkhalsomyndigheten.se (19/521, 3.6%). Organizations other than the WHO did not actively use scientific articles.

Table 6 shows the country distribution for COVID-19 variant citations. For COVID-19 variant citations, when intergovernmental organizations were included, Switzerland ranked first, followed by the Netherlands, the United Kingdom, and the United States. On the other hand, when intergovernmental organizations were excluded, the Netherlands ranked first, followed by the United Kingdom, Australia, and the United States. As mentioned above, the rapid increase in new COVID-19 cases affected these countries. However, the number of citations was not as high as those for COVID-19 vaccines, and the appearance of European countries was greatly reduced.

**Table 3:** Website domain distribution for COVID-19 vaccine citations.

Category and website			Value (N=1900), n (%)
**Intergovernmental organizations**			1453 (76.5)
	who.int	1282 (67.5)	
	worldbank.org	54 (2.8)	
	europa.eu	45 (2.4)	
	fao.org	33 (1.7)	
	au.int	18 (0.9)	
	oecd-ilibrary.org	10 (0.5)	
	iadb.org	6 (0.3)	
	amnesty.org	2 (0.1)	
	wmo.int	2 (0.1)	
	unicef.org	1 (0.1)	
**National and domestic governmental organizations**			333 (17.5)
	rivm.nl	73 (3.8)	
	folkhalsomyndighete.nl	50 (2.6)	
	health.gov.au	34 (1.8)	
	gov.scot	30 (1.6)	
	files.parliament.uk	29 (1.5)	
	org.au	23 (1.2)	
	cdc.gov	20 (1.1)	
	rijksoverheid.nl	19 (1.0)	
	publishing.service.co.uk	17 (0.9)	
	congress.gov	12 (0.6)	
	regjeringen.no	8 (0.4)	
	nice.org.uk	6 (0.3)	
	awmf.org	5 (0.3)	
	idsa.in	4 (0.2)	
	officielebekendmaki.nl	3 (0.2)	
**Nongovernmental organizations (think tanks and academic institutions)**			114 (6.0)
	urban.org	20 (1.1)	
	interacademies.org	18 (0.9)	
	nber.org	15 (0.8)	
	turing.ac.uk	15 (0.8)	
	brookings.edu	14 (0.7)	
	africaportal.org	8 (0.4)	
	rand.org	8 (0.4)	
	americanactionforum.org	6 (0.3)	
	americalatinagenera.org	2 (0.1)	
	bruegel.org	2 (0.1)	
	csis.org	2 (0.1)	
	realinstitutoelcano.org	2 (0.1)	
	georgetown.edu	1 (0.1)	
	mdpi.com	1 (0.1)	

**Table 4:** Country distribution for COVID-19 vaccine citations.

Intergovernmental organization status and region				Value, n (%)
**Intergovernmental organizations included (n=1900)**				
	**United States and Europe**			1811 (95.3)
		Switzerland	1285 (67.6)	
		Netherlands	190 (10.0)	
		United States	161 (8.5)	
		United Kingdom	97 (5.1)	
		Italy	51 (2.7)	
		France	10 (0.5)	
		Norway	8 (0.4)	
		Germany	5 (0.3)	
		Brussel	2 (0.1)	
		Spain	2 (0.1)	
	**Others**			89 (4.7)
		Australia	57 (3.0)	
		Ethiopia	18 (0.9)	
		South Africa	8 (0.4)	
		India	4 (0.2)	
		Panama	2 (0.1)	
**Intergovernmental organizations excluded (n=447)**				
	Netherlands			145 (32.4)
	United States			98 (21.9)
	United Kingdom			97 (21.7)
	Australia			57 (12.8)
	Italy			18 (4.0)
	Norway			8 (1.8)
	South Africa			8 (1.8)
	Germany			5 (1.1)
	India			4 (0.9)
	Panama			2 (0.4)
	Belgium			2 (0.4)
	Spain			2 (0.4)
	Switzerland			1 (0.2)

**Table 5:** Website and domain distribution for COVID-19 variant citations.

Category and website			Value (N=521), n (%)
**Intergovernmental organizations**			460 (88.3)
	who.int	446 (85.6)	
	publishing.service.gov.uk	6 (1.2)	
	worldbank.org	3 (0.6)	
	au.int	2 (0.4)	
	oecd-ilibrary.org	2 (0.4)	
	iadb.org	1 (0.2)	
**National and domestic governmental organizations**			48 (9.2)
	folkhalsomyndighete.nl	19 (3.6)	
	rivm.nl	15 (2.9)	
	publishing.service.gov.uk	6 (1.2)	
	health.gov.au	4 (0.8)	
	parliament.uk	2 (0.4)	
	gov.scot	1 (0.2)	
	rijksoverheid.nl	1 (0.2)	
**Nongovernmental organizations (think tanks and academic institutions)**			13 (2.5)
	interacademies.org	3 (0.6)	
	rand.org	3 (0.6)	
	brookings.edu	2 (0.4)	
	nber.org	2 (0.4)	
	apo.org.au	1 (0.2)	
	awmf.org	1 (0.2)	
	urban.org	1 (0.2)	

**Table 6:** Country distribution for COVID-19 variant citations.

Intergovernmental organization status and country			Value, n (%)
**Intergovernmental organizations included (n=515)**			
	Switzerland	446 (85.6)	
	Netherlands	35 (6.7)	
	United Kingdom	16 (3.1)	
	United States	11 (2.1)	
	Australia	5 (1.0)	
	Italy	3 (0.6)	
	Ethiopia	2 (0.4)	
	France	2 (0.4)	
	Germany	1 (0.2)	
**Intergovernmental organizations excluded (n=** **64** **)**			
	Netherlands	35 (54.7)	
	United Kingdom	10 (15.6)	
	Australia	8 (12.5)	
	United States	7 (10.9)	
	Italy	3 (4.7)	
	Germany	1 (1.6)	

### Network of COVID-Related Research Citations

**Figure 2:**
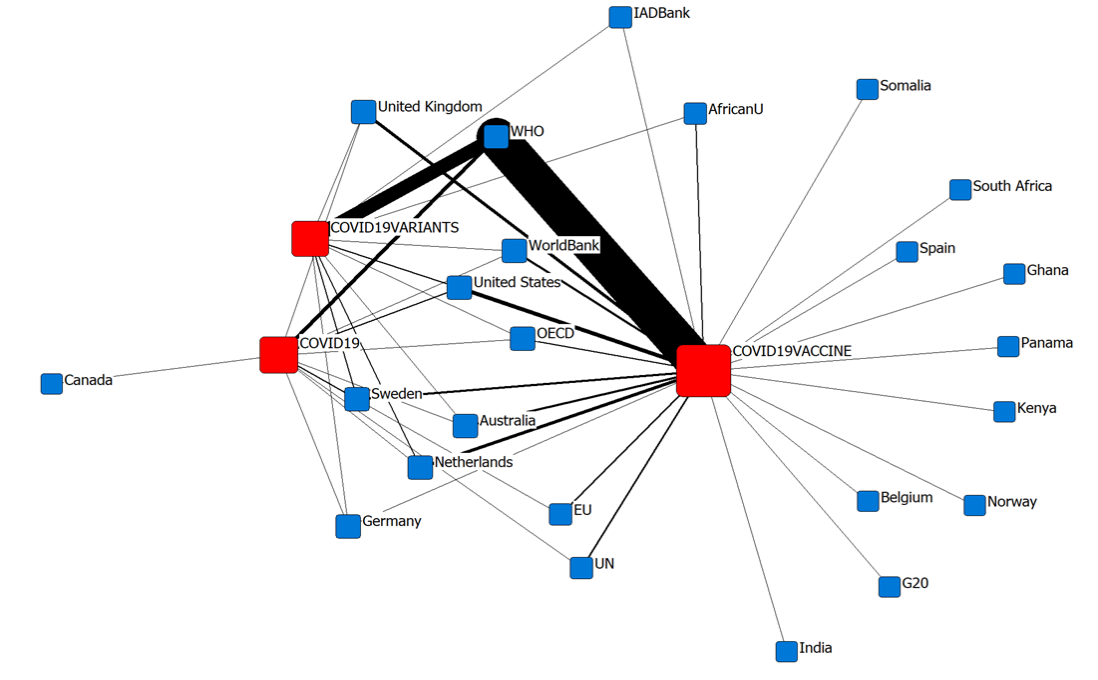
Global network map of COVID-19–related research topics. AfricanU: African Union; EU: European Union; IADBank: Inter-American Development Bank; OECD: Organization for Economic Co-operation and Development; UN: United Nations; WHO: World Health Organization.

Figure 2 depicts a global network map created with the help of a graphical theoretic layout made available through UCINET-NetDraw [21]. The thickness of the lines connecting scientific themes and countries increased as the number of scientific topics per policy report issued in the countries increased.

COVID-19 vaccine was the scientific topic that appeared in most policy papers, followed by COVID-19 variants. In policy documents from early 2022, COVID-19 was the least commonly cited research topic. The size of the squares representing each topic grew in proportion to the number of times it appeared. The link to the WHO is easily distinguishable in Figure 2. Countries, such as Australia, the Netherlands, the United States, and the United Kingdom, showed strong ties to the key term COVID-19 vaccine, along with other intergovernmental organizations. In terms of country diversity, COVID-19 vaccine was the most famous topic among many countries other than those in North America and Europe. In particular, the vested interest of African countries appeared to be strong.

Tables 7 and 8 summarize the multiple network centrality measures of scientific topics and countries. In terms of these measures, it was noted that the topic of COVID-19 vaccine was more central than the other 2 topics of COVID-19 variants and COVID-19 itself.

Through the network measures, the use of scientific information by intergovernmental organizations was clearly visible. Moreover, the absence of Asian countries was noticeable. This may be due to the way government reports are stored, for example, use of JavaScript, inability to use a simple web scrapper, or language issues. Further research would help in understanding this imbalance.

**Table 7:** Country and organization centralities in the COVID-19 research information network (normalized).

Number	Country/organization	Degree	2-local eigenvector	Eigenvector	Coreness
1	World Health Organization	70.615	2689396	0.988	0.115
2	United States	5.500	0.531	0.087	0.115
3	Netherlands	4.423	189863	0.069	0.115
4	United Kingdom	4.269	0.531	0.066	0.115
5	Sweden	3.077	106817	0.039	0.115
6	Australia	2.654	112294	0.041	0.115
7	World Bank	2.346	105063	0.038	0.115
8	European Union	1.769	84144	0.030	0.077
9	United Nations	1.615	73038	0.026	0.077
10	African Union	0.808	37225	0.013	0.077
11	Organization for Economic Co-operation and Development	0.500	20242	0.007	0.115
12	Germany	0.308	12107	0.004	0.115
13	Norway	0.308	15240	0.005	0.038
14	Inter-American Development Bank	0.269	11945	0.004	0.077
15	India	0.154	7620	0.003	0.038
16	Belgium	0.115	5715	0.002	0.038
17	G20	0.115	5715	0.002	0.038
18	South Africa	0.115	0.473	0.002	0.038
19	Ghana	0.077	3810	0.001	0.038
20	Panama	0.077	3810	0.001	0.038
21	Spain	0.077	3810	0.001	0.038
22	Canada	0.038	162	0.000	0.038
23	Kenya	0.038	1905	0.001	0.038
24	Somalia	0.038	1905	0.001	0.038

**Table 8:** Topic centralities in the COVID-19 research information network.

Research topic	Degree	2-local eigenvector	Eigenvector
COVID-19 vaccine	73.269	2408033	0.943
COVID-19 variants	19.808	825399	0.323
COVID-19	6.231	203066	0.079

## Discussion

### Principal Findings

According to the findings, the global scientific network ecology surrounding the COVID-19 pandemic was found to contain distinctive kinds of links that were concentrated around the WHO. In articulating global health policy and coordinating global governance in the early stages of disease diffusion [24,25], the WHO was indeed at the center of the global response system. In addition, based on an analysis of the various kinds of citation ties, Western countries were found to operate effectively in terms of network construction. The preeminent standing of COVID-19 vaccines demonstrates that nation-states align with global authority regardless of the national context in which they operate.

As citation analysis is a promising area of assessing scientific influence, the selection of the networking practices of citations by policy agencies can serve as a proxy signal for the networking strategy that can be used for the next possible pandemic [26]. In other words, a top-down process directed by a global health authority and a catch up from each country could result in a structural change, which may be substantially modified by the altmetrics-supplied research collaboration network.

The findings of this study have a number of significant ramifications. Analyzing policy document publication citation practices is a new research field, which is currently being explored [27]. For instance, the typical number of citations to scholarly works found in COVID-19 policy documents has not been thoroughly investigated. Furthermore, present research indicates that policy document publishers used academic references in a unique way during the pandemic period. The findings, for instance, identified the scientific articles and journals most frequently cited in WHO policy documents. Hence, the organization of citation networks serves as a stand-in for the policy knowledge base, which is an essential component in the process of mitigating potential health hazards. It is likely that the widespread dissemination of COVID-19 might have been accelerated as a result of the delayed recognition of scientific and research information in the public health and government sectors. The increase in the use of preprint scholarly articles highlights the urgent need for COVID-19 scientific information [28]. This is evinced by the fact that each international organization and nation state has its own system for referencing scholarly sources within policy contexts. This is particularly important for developing countries, such as Iran, where policy resources are insufficient for public health management [29]. The increased need for evidence-based policy decisions shows a growing interest in the results of scientific studies and research, particularly when applied to situations involving risk reduction.

Citations from policy websites may also serve as crucial agenda-setting signals for mass media sources that aggregate news and develop country-level trends related to COVID-19 prevention. Although there may be a convergence of research, policy, and the media, the cooperation between these 3 institutional entities has not been sufficiently studied [30]. In other words, this condition signifies a decentralized information transfer toward a more unified health governance in order to establish a solid knowledge base.

### Limitations

From a methodological standpoint, this research has some issues and limitations. This study was not intended to assess the success of scientific research on pandemics in academic settings or in the broader community. Therefore, the number of citations from authors of policy texts should not be viewed as a measure of the impact of the research. In a similar vein, the disparity in centrality measures between policy authors in citation networks does not imply that these authors are ranked differently. The variance, on the other hand, illuminates the extent to which policy document authors are connected to the scientific and research communities during the COVID-19 pandemic. In addition, it should be noted that articles and journals with a high number of citations should not be considered the most credible sources for research findings or publication outlets. This is due to the fact that a large number of citations is not necessarily indicative of a reliable source. The establishment of systematic citation links between scientific research and health policy texts signifies the development of a communication network in addition to the dissemination of information.

### Conclusion

The goal of this study was to find out how institutions view the quality of research information, as well as whether they are aware of COVID-19 and the emergency effect that its vaccine has on the prevention of the global pandemic. We found that who.int was the source of majority of the websites and domains containing scientific documents on COVID-19 variants. This demonstrates that who.int is actively seeking and sharing information in the COVID-19 research community. Furthermore, the “COVID-19 vaccine” key term had the most extensive connections in terms of degree centrality, as well as 2-local and eigenvector centralities. The countries that were most actively sought after and tied with one another were the Netherlands, the United States, the United Kingdom, and Australia. Although developing countries obtained COVID-19 vaccine information more quickly and with greater ease, their marginal position in the global network indicated that they were isolated from enriched COVID-19 pandemic content. As developing countries are predominantly consumers of pandemic content as opposed to active producers of information, they are isolated from the research and development activities associated with COVID-19 [31], which in turn can impact their connections to other information sources from established countries. Additionally, this may be due to the fact that the prioritization of global research may not be particularly suited to developing nations. The dissemination of enriched COVID-19 content may prioritize regions with enhanced connectivity and more significant resources, resulting in the concentration of data within particular networks. This can lead to developing nations receiving less attention and representation in the global influx of pandemic-enriched content.

The entire structure of the COVID-19 research information network regarding website distribution and centrality included in the study demonstrated the need for emergency services for scientific information in global public health governance. A number of methods that facilitate this could be suggested, especially for developing countries. Among them, one approach is to expand the network of partnerships and knowledge exchange with developed nations. Similar to COVAX (COVID-19 Vaccines Global Access), the alliance for the exchange of knowledge and information can induce and position developing countries in the center of a global network with enriched pandemic content. Another approach is to increase alliances and networks between developing nations, which can increase the significance of their responsibilities in the global information flow network.

This study is notable because it is one of the few altmetric studies that looked at how international policy institutions in the health sector used the internet for information seeking and sharing from the perspective of actor-document networks.

We were unable to determine whether the quarantine and lockdown stances on COVID-19 of specific nations eventually influenced their network positions or the manner in which research information was provided to them. Additional research should be conducted using qualitative methodologies to analyze the citation circumstances of policy research. For example, it must be evaluated whether policy agencies established their own autonomous cluster in order to pool resources and collaborate. Furthermore, we did not investigate the extent to which policy institutions working with smaller or developing nations aided in enhancing citizen involvement with the COVID-19 issues at hand. Furthermore, we were unable to detect structural movement in the online altmetric network between several time points that were analyzed.

## Appendix

Multimedia Appendix 1 COVID-19 new case trends for the Netherlands, the United Kingdom, the United States, and Australia.

## Abbreviations

ccTLD: country code top-level domain
WHO: World Health Organization
