# Supplementary figures and images for: Global COVID-19 Policy Engagement With Scientific Research Information: Altmetric Data Study
Source: J Med Internet Res. 2023 Jun 29;25:e46328. doi: 10.2196/46328 (PMC10365591; doi:10.2196/46328)

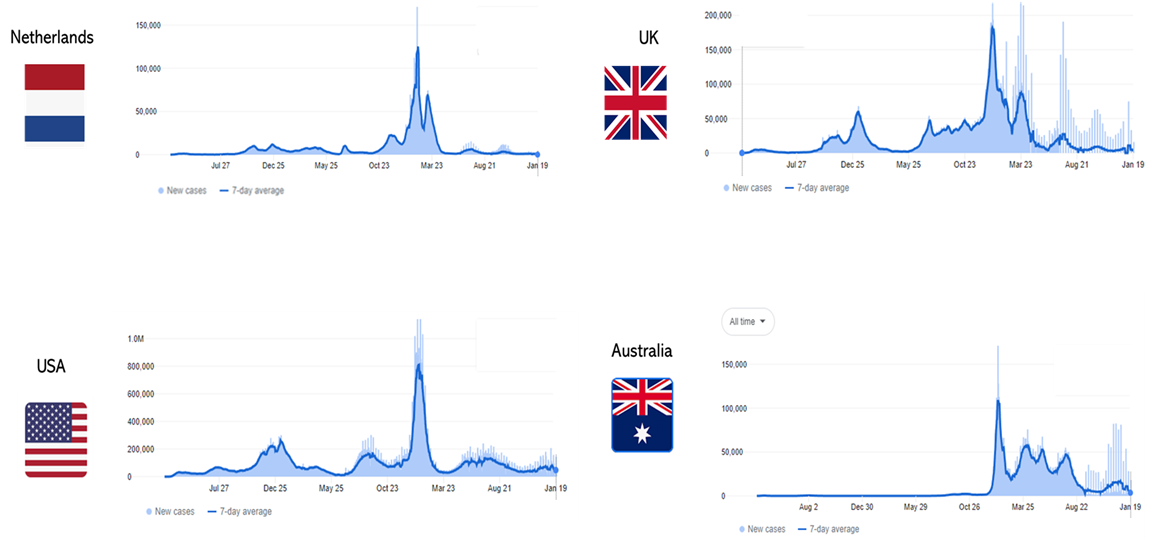

Supplement: Multimedia Appendix 1 [file jmir_v25i1e46328_app1.png]
